# *Lgals9* deficiency ameliorates obesity by modulating redox state of PRDX2

**DOI:** 10.1038/s41598-021-85080-1

**Published:** 2021-03-16

**Authors:** Tomokazu Nunoue, Satoshi Yamaguchi, Sanae Teshigawara, Akihiro Katayama, Atsuko Nakatsuka, Jun Eguchi, Toshiro Niki, Jun Wada

**Affiliations:** 1 Department of Nephrology, Rheumatology, Endocrinology and Metabolism, Okayama University Graduate School of Medicine, Dentistry and Pharmaceutical Sciences, 2-5-1 Shikata-cho, Kita-ku, Okayama, 700-8558 Japan; 2 Department of Immunology, Kagawa University, Takamatsu, Kagawa Japan

**Keywords:** Experimental models of disease, Molecular biology, Health care, Medical research, Diseases, Metabolic disorders

## Abstract

The adipose tissue is regarded as an endocrine organ and secretes bioactive adipokines modulating chronic inflammation and oxidative stress in obesity. Gal-9 is secreted out upon cell injuries, interacts with T-cell immunoglobulin-3 (Tim-3) and induces apoptosis in activated Th1 cells. Gal-9 also binds to protein disulfide isomerase (PDI), maintains PDI on surface of T cells, and increases free thiols in the disulfide/thiol cycles. To explore the molecular mechanism of obesity, we investigated Gal-9^−/−^ and Gal-9^wt/wt^ C57BL/6J mice fed with high fat-high sucrose (HFHS) chow. Gal-9^−/−^ mice were resistant to diet-induced obesity associated with reduction of epididymal and mesenteric fat tissues and improved glucose tolerance compared with Gal-9^wt/wt^ mice. However, the number of M1, M2 macrophages, and M1/M2 ratio in epididymal fat were unaltered. Under HFHS chow, Gal-9^−/−^ mice receiving Gal-9^−/−^ or Gal-9^wt/wt^ bone marrow-derived cells (BMCs) demonstrated significantly lower body weight compared with Gal-9^wt/wt^ mice receiving Gal-9^−/−^ BMCs. We identified the binding between Gal-9 and peroxiredoxin-2 (PRDX2) in sugar chain-independent manner by nanoLC-MS/MS, immunoprecipitation, and pull-down assay. In 3T3L1 adipocytes, Gal-9 knockdown shifts PRDX2 monomer (reduced form) dominant from PRDX2 dimer (oxidized form) under oxidative stress with H_2_O_2_. The inhibition of Gal-9 in adipocytes may be a new therapeutic approach targeting the oxidative stress and subsequent glucose intolerance in obesity.

## Introduction

The obesity is now pandemic in worldwide and the data from WHO demonstrated that more than 1.9 billion adults were overweight and over 650 million obese in 2016 (https://www.who.int/news-room/fact-sheets/detail/obesity-and-overweight). It substantially increases the risk of diseases such as type 2 diabetes, fatty liver disease, dyslipidemia and hypertension^1^. The adipose tissue has been regarded as an endocrine organ and it secretes bioactive adipokines leading to low grade chronic inflammation and amplification of oxidative stress^2^. In the obese patients, the chronic inflammation is demonstrated by the elevation of high-sensitive C-reactive protein^3^, interleukin (IL)-6^3^, IL-10, and tumor necrosis factor α (TNF-α)^4^, while the oxidative stress revealed by decreased antioxidant enzymes (catalase, glutathione peroxidase, superoxide dismutase)^5^ or increased levels of malondialdehyde^3,5^, urinary 8-hydroxy-2′-deoxyguanosine^6^ and lipid peroxidation markers (8-iso-prostaglandin F2α)^7^. By targeting inflammation and oxidative stress in obese patients, the interventions with foods and supplements in a randomized controlled trial have been conducted, such as Mediterranean diet with extra virgin olive oil^6^, pigmented rice^8^, Baru almonds^5^, and curcumin^3^. Although life-style modifications and intervention with foods and supplements have been vigorously attempted, the mechanism for the inflammation and oxidative stress in obesity is not fully explored.

Galectin-9 (Gal-9) is consisted of N- and C-terminal carbohydrate binding domains, which recognize β-galactoside sugar structure^9,10^. Gal-9 is found to be involved in intracellular and extracellular processes, such as glycoprotein trafficking, cell–cell or cell-extracellular matrix interaction, signal transduction, immune response, development and oncogenesis^11^. Gal-9 is induced by various stimuli such as interferon-γ, IL-1β, toll-like receptor agonists, several different viruses^12,13^, and Leishmania parasites^14^. Gal-9 is secreted out from cytoplasm to extracellular space in non-classical secretion pathway as damage-associated molecular patterns (DAMPs) or pathogen-associated molecular patterns (PAMPs)^14–16^. Secreted Gal-9 interacts as a ligand with N-glycans covalently attached to the surface of T-cell immunoglobulin-3 (Tim-3)^17^. Gal-9 triggers a series of events like calcium mobilization, calpain and caspase-1 activation and drives the Th1 cells to apoptosis. The injection of recombinant Gal-9 induced the apoptosis of T cells and ameliorated the diseases such as nephrotoxic nephritis^18^, type 1 diabetes^19^, and systemic lupus erythematosus (SLE)^20^. Tim-3 and Gal-9 are also targets for the immune checkpoint inhibition immunotherapies for cancers^21^. Although the roles of Gal-9 in immune response such as the apoptotic potential against Tim3 + Th1, Th17^22^, Tim-3 + NKT^23^, and CD8^24^ cells are well-investigated, the functional role of Gal-9 in oxidative stress and redox status is totally unexplored. Recently, Gal-9 is reported to retain protein disulfide isomerase (PDI) on cell surface of T cells by binding to O-glycans on PDI. PDI creates disulfide bonds in nascent protein and shifts the disulfide/thiol equilibrium on the T cell surface^25^.

The adipose tissues in obesity represent chronic inflammation characterized by Th1/M1-macrophage dominant *versus* Th2/M2 cells^26^. In addition, the oxidative stress is critically involved of development of metabolic syndrome phenotype in obesity^27^. To explore the molecular mechanism of inflammation and oxidative stress in obesity, we investigated the Lgals9^tm1Glp^/Lgals9^tm1Glp^ (Gal-9^−/−^) C57BL/6J mice fed with high fat-high sucrose diet (HFHS) and analyzed the phenotypes. Here, we demonstrate Gal-9^−/−^ C57BL/6J mice are resistant to diet-induced obesity independent of bone marrow-derived cells. We also show that Gal-9 binds to peroxiredoxin-2 (PRDX2) and Gal-9 knockdown shifts PRDX2 monomer (reduced form) dominant against PRDX2 dimer (oxidized form) under oxidative stress.

## Results

### Gal-9 deficiency contributes the resistance to obesity

**Figure 1:**
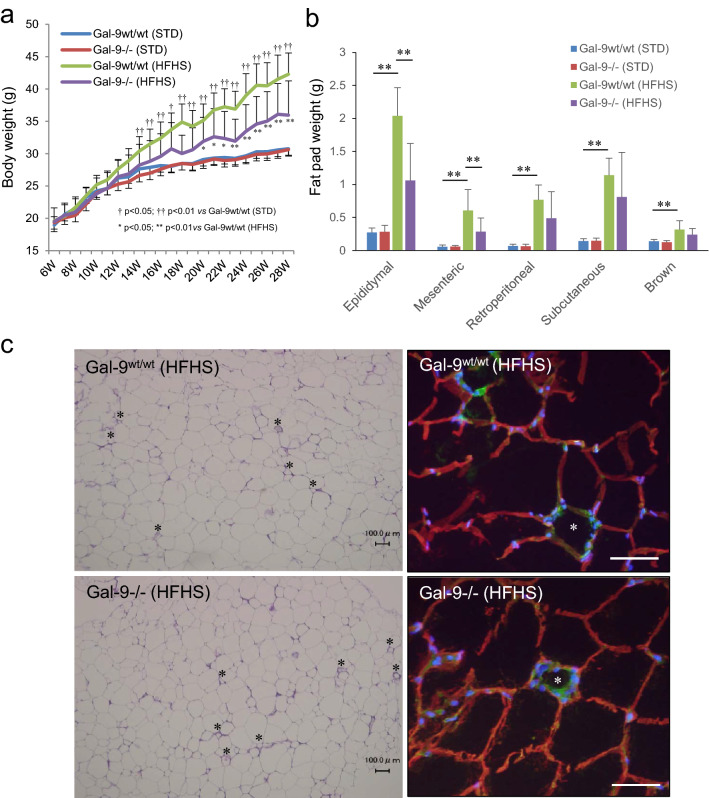
Body and fat pad weights and histology of epididymal adipose tissues. (**a**) Body weight in Gal-9^wt/wt^ and Gal-9^−/−^ mice fed with standard (STD) and high fat-high sucrose (HFHS) chow. Gal-9^wt/wt^ (STD), n = 9; Gal-9^−/−^ (STD), n = 10, Gal-9^wt/wt^ (HFHS), n = 9; Gal-9^−/−^ (HFHS), n = 12. *, *p* < 0.05; **, *p* < 0.01 [Gal-9^wt/wt^ (HFHS) vs*. *Gal-9^−/−^ (HFHS)]; †, *p* < 0.05; ††, *p* < 0.01 [Gal-9^wt/wt^ (STD) vs*.* Gal-9^wt/wt^ (HFHS)]. One-way ANOVA with Tukey–Kramer. (**b**) Fat pad weight. Gal-9^wt/wt^ (STD), n = 9; Gal-9^−/−^ (STD), n = 8, Gal-9^wt/wt^ (HFHS), n = 8; Gal-9^−/−^ (HFHS), n = 9. *, *p* < 0.05; **, *p* < 0.01. One-way ANOVA with Tukey–Kramer. **c**. PAS stain and immunofluorescence study. *, Crown-like structures; Green, F4/80; Red, Perilipin; Blue DAPI. Bar = 100 μm.

**Figure 2:**
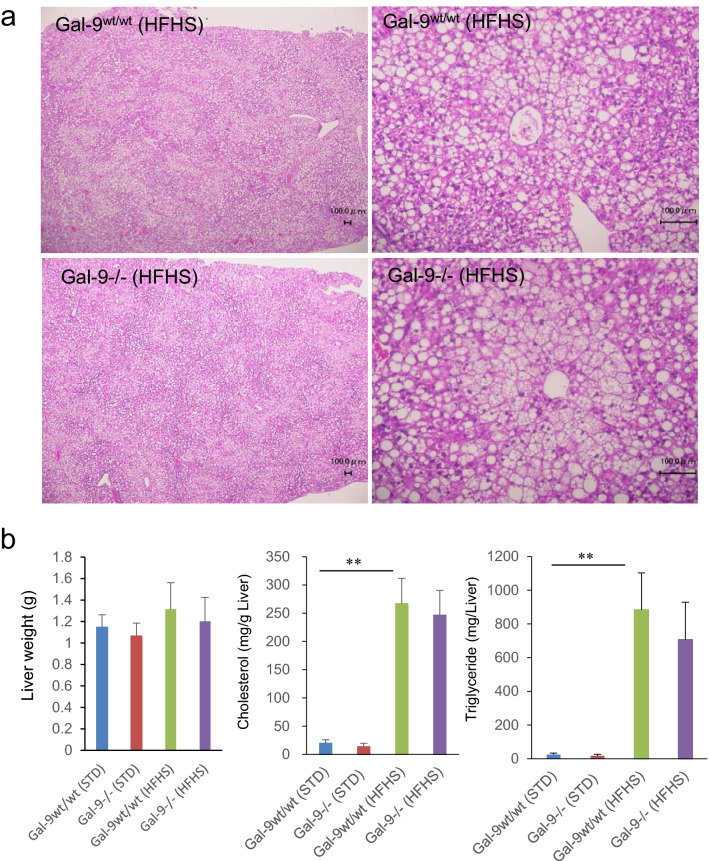
Histology of the liver, weight, cholesterol, and triglyceride contents. (**a**) PAS staining of the liver. There are no differences in lipid droplets in the liver. (**b**) Liver weight, cholesterol and triglyceride contents. Gal-9^wt/wt^ (STD), n = 8; Gal-9^−/−^ (STD), n = 5, Gal-9^wt/wt^ (HFHS), n = 7; Gal-9^−/−^ (HFHS), n = 5 in liver weight. Gal-9^wt/wt^ (STD), n = 5; Gal-9^−/−^ (STD), n = 6, Gal-9^wt/wt^ (HFHS), n = 5; Gal-9^−/−^ (HFHS), n = 6 in cholesterol and triglyceride contents. *, *p* < 0.05; **, *p* < 0.01. One-way ANOVA with Tukey–Kramer.

Under standard (STD) chow, there was no difference in body weights between Gal-9^−/−^ (29.4 ± 2.4 g) and Gal-9^wt/wt^ mice (30.8 ± 1.4 g) at 28 weeks (*p* = 0.999) (Fig. 1a). After the initiation of HFHS chow, Gal-9^−/−^ mice gained less body weight (41.2 ± 4.5 g) compared with Gal-9^wt/wt^ mice (43.5 ± 0.9 g) and it was statistically significant at 28 weeks of age (*p* = 2.16 × 10^–7^) (Fig. 1a). Under HFHS chow at 28 weeks of age, the fat pad weights of visceral white adipose tissues (WATs) were also lower in Gal-9^−/−^ mice compared with Gal-9^wt/wt^ mice in epididymal (1.13 ± 0.52 g vs*.* 2.03 ± 0.42 g, *p* = 2.2 × 10^–4^), mesenteric (0.30 ± 0.21 g vs*.* 0.60 ± 0.31 g, *p* = 0.007), retroperitoneal (0.54 ± 0.40 g vs*.* 0.77 ± 0.22 g, *p* = 0.092), and subdermal (0.89 ± 0.68 g vs*.* 1.14 ± 0.26 g, *p* = 0.273) WATs (Fig. 1b), respectively. Although the mass of WATs was reduced in in Gal-9^−/−^ mice fed with HFHS chow, the size of adipocytes and frequency of crown-like structure were not altered in Gal-9^−/−^ mice compared with Gal-9^wt/wt^ mice (Fig. 1c). In contrast to WATs, there were no apparent changes in liver weight, size and distribution of lipid droplets in hepatocytes, tissue cholesterol and triglyceride contents in liver tissues in Gal-9^−/−^ mice fed with HFHS chow (Fig. 2a,b).

### ***Glucose and lipid metabolism are improved in Gal-9***^−/−^*** mice***

**Figure 3:**
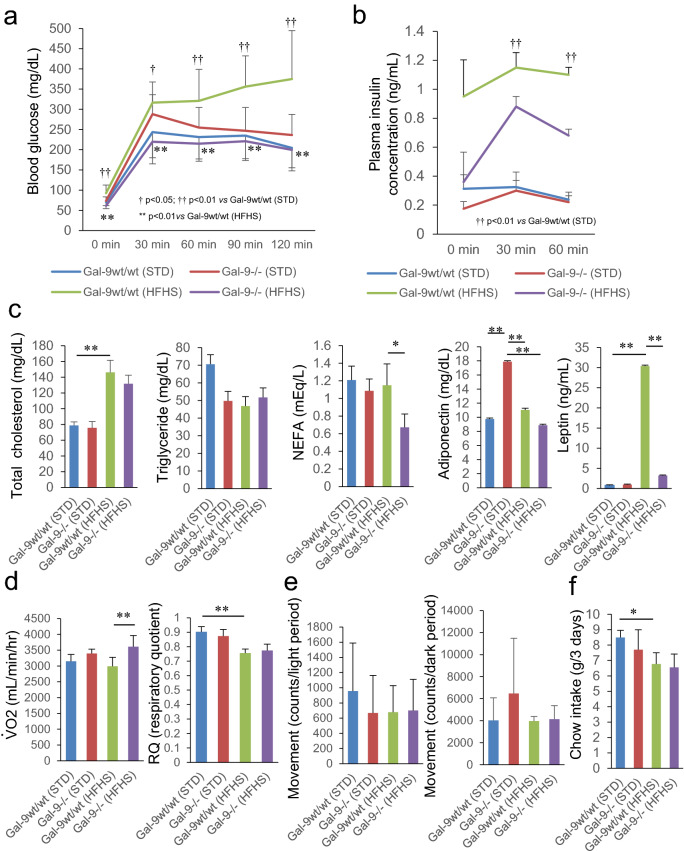
Glucose, lipid metabolism, and energy expenditures. (**a**) Blood glucose levels in intraperitoneal glucose tolerance tests (IPGTT; 1 mg/g weight) at 20–22 weeks of age. Gal-9^wt/wt^ (STD), n = 11; Gal-9^−/−^ (STD), n = 12, Gal-9^wt/wt^ (HFHS), n = 11; Gal-9^−/−^ (HFHS), n = 13. *, *p* < 0.05; **, *p* < 0.01 [Gal-9^wt/wt^ (HFHS) vs*.* Gal-9^−/−^ (HFHS)]; †, *p* < 0.05; ††, *p* < 0.01 [Gal-9^wt/wt^ (STD) vs*.* Gal-9^wt/wt^ (HFHS)]. One-way ANOVA with Tukey–Kramer. (**b**) Plasma insulin concentration in intraperitoneal glucose tolerance test. Gal-9^wt/wt^ (STD), n = 8; Gal-9^−/−^ (STD), n = 5, Gal-9^wt/wt^ (HFHS), n = 7; Gal-9^−/−^ (HFHS), n = 5. †, *p* < 0.05; ††, *p* < 0.01 [Gal-9^wt/wt^ (STD) vs*.* Gal-9^wt/wt^ (HFHS)]. Kruskal–Wallis test with Bonferroni correction. (**c**) Serum levels of cholesterol, triglyceride, non-esterified fatty acid (NEFA), adiponectin and leptin. Gal-9^wt/wt^ (STD), n = 4; Gal-9^−/−^ (STD), n = 4, Gal-9^wt/wt^ (HFHS), n = 4; Gal-9^−/−^ (HFHS), n = 4. *, *p* < 0.05; **, *p* < 0.01. (**d**) Oxygen consumption rate ($\dot{V}$˙O_2_) and respiratory quotient (RQ). Gal-9^wt/wt^ (STD), n = 3; Gal-9^−/−^ (STD), n = 6, Gal-9^wt/wt^ (HFHS), n = 4; Gal-9^−/−^ (HFHS), n = 6. **, *p* < 0.01. (**e**) Locomotor activities during light and dark periods. Gal-9^wt/wt^ (STD), n = 3; Gal-9^−/−^ (STD), n = 4, Gal-9^wt/wt^ (HFHS), n = 4; Gal-9^−/−^ (HFHS), n = 5. (**f**) Chow intake. Gal-9^wt/wt^ (STD), n = 3; Gal-9^−/−^ (STD), n = 5, Gal-9^wt/wt^ (HFHS), n = 4; Gal-9^−/−^ (HFHS), n = 7. One-way ANOVA with Tukey–Kramer (**c**–**f**).

The intraperitoneal glucose tolerance tests (IPGTT) were performed during 20–22 weeks of age. The IPGTT demonstrated that blood glucose levels were significantly lower in Gal-9^−/−^ mice fed with HFHS chow compared with Gal-9^wt/wt^ mice in the fasting and during the test period (Fig. 3a). During IPGTT, plasma insulin levels revealed lower tendency in Gal-9^−/−^ mice compared with Gal-9^wt/wt^ mice fed with HFHS chow, although it was not statistically significant (Fig. 3b). At 28 weeks of age, fasting serum levels of total cholesterol and triglyceride demonstrated no changes in Gal-9^−/−^ mice compared with Gal-9^wt/wt^ mice fed with HFHS chow (Fig. 3c). However, non-esterified free fatty acids (NEFA) (0.67 ± 0.15 vs*.* 1.15 ± 0.24 mEq/L) and serum leptin levels (3.23 ± 4.86 vs*.* 30.4 ± 6.80 ng/mL, *p* = 5.0 × 10^–6^) were significantly reduced in Gal-9^−/−^ mice compared with Gal-9^wt/wt^ mice fed with HFHS chow (Fig. 3c). Interestingly, *Lgals9* deficiency exhibited beneficial effects of up-regulated serum levels of adiponectin under STD chow; however, such effects were cancelled under HFHS chow (Fig. 3c). To investigate the energy expenditure, oxygen consumption rate ($\dot{V}$˙O_2_) and respiratory quotient (RQ) were measured. $\dot{V}$˙O_2_ was significantly increased in Gal-9^−/−^ mice (3613 ± 350 mL/min/hr) compared with Gal-9^wt/wt^ mice (2992 ± 283 mL/min/hr) fed with HFHS chow (*p* = 0.011) RQ remained in similar levels in both Gal-9^−/−^ mice (0.77 ± 0.04) and Gal-9^wt/wt^ mice (0.76 ± 0.03) fed with HFHS chow (Fig. 3d). Locomotor activities, the intake of STD and HFHS chow were also similar in both groups (Fig. 3e,f).

### ***Contribution of inflammatory cells for the protection of obesity in Gal-9***^−/−^*** mice is minimal***

**Figure 4:**
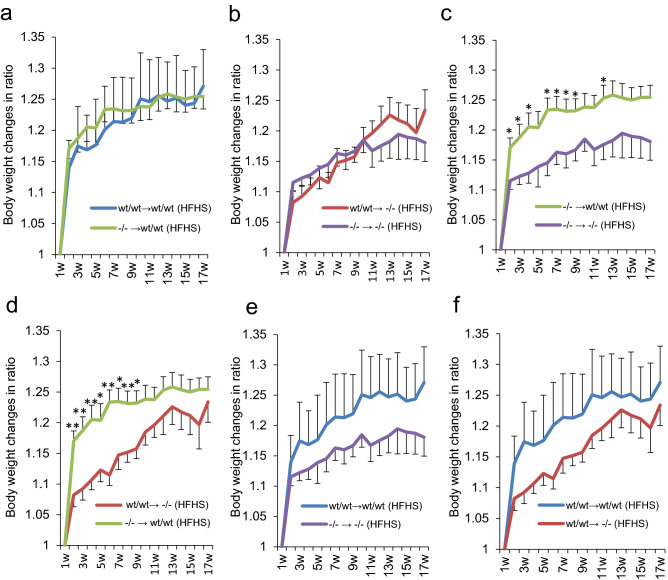
Body weight gain ratio in mice receiving bone marrow transplantation and fed with HFHS chow. The body weight at baseline is set as a reference. (**a**) WT → WT (n = 6), Gal-9^wt/wt^ (donors) and Gal-9^wt/wt^ (recipients); KO → WT (n = 8), Gal-9^−/−^ (donors) and Gal-9^wt/wt^ (recipients). (**b**) WT → KO (n = 7), Gal-9^wt/wt^ (donors) and Gal-9^−/−^ (recipients); KO → KO (n = 6), Gal-9^−/−^ (donors) and Gal-9^−/−^ (recipients). (**c**) KO → WT (n = 8), Gal-9^−/−^ (donors) and Gal-9^wt/wt^ (recipients); KO → KO (n = 6), Gal-9^−/−^ (donors) and Gal-9^−/−^ (recipients). (**d**) WT → KO (n = 7), Gal-9^wt/wt^ (donors) and Gal-9^−/−^ (recipients); KO → WT (n = 8), Gal-9^−/−^ (donors) and Gal-9^wt/wt^ (recipients). (**e**) WT → WT (n = 6), Gal-9^wt/wt^ (donors) and Gal-9^wt/wt^ (recipients); KO → KO (n = 6), Gal-9^−/−^ (donors) and Gal-9^−/−^ (recipients). (**f**) WT → WT (n = 6), Gal-9^wt/wt^ (donors) and Gal-9^wt/wt^ (recipients); WT → KO (n = 7), Gal-9^wt/wt^ (donors) and Gal-9^−/−^ (recipients). *,* p* < 0.05; **, *p* < 0.01. Two-pair comparisons by Student’s *t* test.

Since the injection of recombinant Gal-9 into mice induces the apoptosis of activated T-cell immunoglobulin mucin-3 (Tim-3) positive Th1 cells, we initially hypothesized that the deficiency of Gal-9 may further shift M1/M2 balance of the adipose tissue macrophages to M1 polarization. First, we investigated the gene expression profile of M1 and M2 macrophage markers using total RNA isolated from whole epididymal adipose tissues. Under HFHS chow, the M1 markers including *Il6*, *Il10*, *Nos2*, and *Tnf* increased except for *Il18*, while M2 markers such *Arg1* and *Ym1* were reduced compared with STD chow groups in both Gal-9^wt/wt^ and Gal-9^−/−^ mice, although statistically significant changes were observed only in *Il6* and *Arg1*. There were no differences in both M1 and M2 markers between Gal-9^wt/wt^ and Gal-9^−/−^ mice fed with HFHS chow (Supplementary Fig. 1a). Next, we investigated the population of M1 and M2 macrophages in stromal vascular fractions (SVF) from epididymal fat pads by FACS analyses. F4/80 + CD11b + /CD45.2 + (macrophages) and CD11c + CD206-/F4/80 + CD11b + (M1 macrophages) increased, while CD11c-CD206 + /F4/80 + CD11b + (M2 macrophages) decreased in both Gal-9^wt/wt^ and Gal-9^−/−^ mice. Again, there were no differences of M1/M2 ratios between Gal-9^wt/wt^ and Gal-9^−/−^ mice fed with HFHS chow (Supplementary Fig. 1b). We next tried to clarify whether the absence of Gal-9 in inflammatory cells or in adipocytes contributed the obesity-resistant phenotypes in Gal-9^−/−^ mice fed with HFHS chow. We performed bone marrow transplantation experiments, and 4–6 weeks old Gal-9^wt/wt^ and Gal-9^−/−^ recipient mice were injected with bone marrow cells (BMCs) derived from Gal-9^wt/wt^ and Gal-9^−/−^ mice. They were fed with HFHS chow and body weight was measured for 16 weeks (Fig. 4). Transplantation of Gal-9^−/−^ BMCs did not reduce the body weight of Gal-9^wt/wt^ mice fed with HFHS chow (Fig. 4a). Furthermore, Gal-9^−/−^ mice fed with HFHS chow did not gain weight by the transfusion of Gal-9^wt/wt^ BMCs (Fig. 4b). The data suggested that the deficiency of Gal-9 in BMCs did not contribute the reduction of body weight in the mice fed with HFHS chow. In contrast, the body weight of Gal-9^−/−^ recipient mice was always lower than Gal-9^wt/wt^ recipient mice fed with HFHS chow under the presence or absence of Gal-9 gene in BMCs (Fig. 4c–f). Under HFHS chow, Gal-9^−/−^ mice receiving Gal-9^−/−^ or Gal-9^wt/wt^ BMCs demonstrated significantly lower body weight compared with Gal-9^wt/wt^ mice receiving Gal-9^−/−^ BMCs (Fig. 4c) or Gal-9^wt/wt^ BMCs (Fig. 4d).

### The identification of Gal-9 interacting proteins in adipocytes

Bone marrow transplantation experiments suggested that the absence of Gal-9 in adipocytes may contribute the resistance to obesity and insulin resistance in Gal-9^−/−^ mice. To identify the Gal-9 interacting proteins in adipocytes, the immunoprecipitated protein complexes by monoclonal anti-Gal-9 antibody or isotype control isolated from epididymal fat pads in Gal-9^wt/wt^ mice were subjected to nanoLC-MS/MS analysis and analyzed by Mascot. Anti-Gal-9 antibody- and isotype control antibody-precipitated proteins and the comparison of exponentially modified protein abundance index (emPAI) values of proteins between 2 samples were listed in Supplementary Table 1a–c. The proteins found only in anti-Gal-9 antibody- and isotype control antibody-precipitated samples are shown in Supplementary Table 1d, e, respectively. Since the absence of intracellular Gal-9 may be involved in the amelioration of obesity, the intracellular proteins were firstly screened. The proteins found only in anti-Gal-9 antibody-precipitated sample were subjected to gene ontology analysis by PANTHER 15.0 (Supplementary Table 2). Gal-9, peroxiredoxin 2 (PRDX2), serine/threonine-protein kinase RIO2, ATP-citrate synthase, cytosolic phospholipase A2 zeta, and serine/threonine-protein kinase WNK1 were identified as cytosolic protein (Supplementary Table 2). The normalized emPAI of PRDX2 is highest and ranked top in these cytosolic proteins. In functional aspects, mitochondrial oxidative stress in adipocytes is known to cause insulin resistance, and 6 mitochondrial proteins, electron transfer flavoprotein subunit alpha (ETFA), ferredoxin-2, 3-ketoacyl-CoA thiolase (ACAA2), long-chain specific acyl-CoA dehydrogenase (ACADVL), acyl-CoA dehydrogenase family member 9 (ACAD9), and carnitine O-palmitoyltransferase 2 (CTP2), were also listed in the proteins found only in anti-Gal-9 antibody-precipitated sample (Supplementary Table 1d and 2).

### Gal-9 binds to PRDX2 and its dimer/monomer ratio is reduced by Gal-9 siRNA

**Figure 5:**
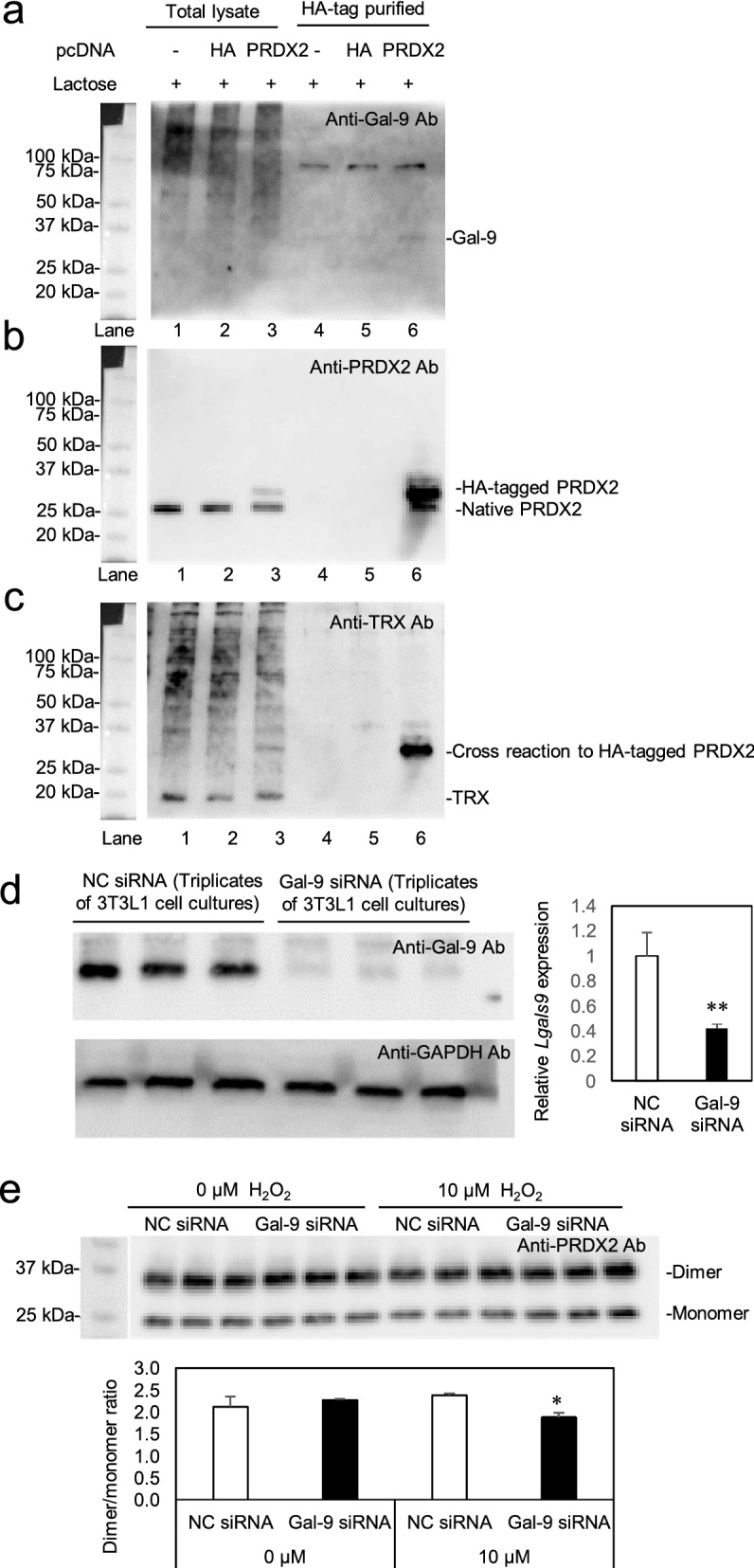
Pull-down assay and Gal-9 siRNA experiments in 3T3L1 cells. (**a**) PRDX2-FLAG-HA-pcDNA3.1 (PRDX2) and FLAG-HA-pcDNA3.1 (HA) were transfected into 3T3L1 cells. In the presence of 0.2 M lactose, the protein complexes were HA-tag purified with Anti-HA tag Beads, and subjected to SDS-PAGE under reducing conditions and Western blot analysis. The membrane was incubated with anti-Gal-9 antibody. (**b**) The membrane was stripped off and incubated with anti-peroxiredoxin 2 (PRDX2) antibody. (**c**) The membranes was again stripped off and incubated with anti-thioredoxin (TRX) antibody. (**d**) 3T3L1 cells were treated with Silencer select Pre-designed siRNA Lgals9 (Gal-9 siRNA) and Silencer select negative control siRNA (NC siRNA) for 40 h. (**e**) After the treatment of 3T3L1 cells with siRNAs, the cells were further cultured in the absence and presence of 10 μM H_2_O_2_ for 20 min. *, *p* < 0.05; **, *p* < 0.01. Two-pair comparisons by Student’s *t* test.

Mitochondria are major source of oxidative stress in adipocytes under obesity and insulin resistance state, and peroxiredoxins are important for antioxidant responses against oxidative stress, we further investigated whether Gal-9 interacts with peroxiredoxins and mitochondrial proteins. Among peroxiredoxins 1, 2, 3, 4, and 5, Gal-9 was detected in the protein complexes immunoprecipitated by anti-PRDX2 antibody in the lysates isolated from epididymal adipose tissues of Gal-9^wt/wt^ mice (Supplementary Fig. 2). Total lysate of 3T3L1 cells without plasmid transfection was applied to Anti-HA tag Beads and Gal-9 was detected by Western blot analysis (Supplementary Fig. 3a, lane 4). However, the binding between Gal-9 and Beads was completely inhibited by the addition of 0.2 M lactose (Fig. 5a, lane 4, Supplementary Fig. 4a). Thus, following experiments were performed in the presence of 0.2 M lactose. PRDX2-FLAG-HA-pcDNA3.1 (PRDX2) and FLAG-HA-pcDNA3.1 (HA) were transfected into 3T3L1 cells, and total cell lysates were subjected to Anti-HA tag Beads isolation and Western blot analysis. Gal-9 and HA-tagged PRDX2 and were detected in protein complexes isolated by Anti-HA tag Beads (Fig. 5b and Supplementary Fig. 4b). Although anti-thioredoxin (TRX) antibody cross-reacted to HA-tagged PRDX2, native TRX was not detected in the HA-tag purified protein complexes (Fig. 5c and Supplementary Fig. 4c). The binding between Gal-9 and PRDX2 is sugar chain-independent, since the addition of 0.2 M lactose, which interrupts the binding between Gal-9 and β-galactoside sugar, did not inhibit the complex formation between Gal-9 and HA-tagged PRDX2.

Two Cysteine residues of PRDX2 form disulfide-linked homodimers upon oxidation by hydroperoxides such as H_2_O_2_, and they were slowly reduced to their monomers by thioredoxin and thioredoxin oxidase system. Thus, PRDX2 dimer/monomer ratio well-reflects cytosolic redox status and higher ratios represent oxidative state in the cytosol. In 3T3L1 cells, Gal-9 siRNA reduced the protein expression of Gal-9 to 42.1 ± 3.2% compared with NC siRNA treated cells (Fig. 5d and Supplementary Fig. 4d). Under the basal states without H_2_O_2_, PRDX2 dimer/monomer ratios were not altered in NC siRNA (2.12 ± 0.23) and Gal-9 siRNA (2.27 ± 0.02) treatments. However, in the presence of 10 μM H_2_O_2_, the dimer/monomer ratios were reduced from 2.38 ± 0.04 to 1.88 ± 0.10 by the treatments with Gal-9 siRNA (Fig. 5e and Supplementary Fig. 4e), suggesting that Gal-9 knockdown shifts the redox state to reducing conditions. We also investigated the oxidative stress signaling pathway such as p38 mitogen-activated protein kinase (p38-MAPK), stress-activated protein kinase (SAPK)/Jun amino terminal kinase (JNK), and p42/p44 mitogen-activated protein kinases [MAPK; extracellular signal-regulated kinase 2/1 (ERK2/1)]. Although the treatment with Gal9-siRNA partially reversed increased phospho/total SAPK/JNK and phospho/total ERK2/1 ratios by the oxidative stress under 10 μM H_2_O_2_, they did not reach the statistical differences (Supplementary Fig. 5).

## Discussion

Adipose tissue macrophages (ATMs) play key roles in the inflammation of adipose tissues and link to the development of insulin resistance in obesity. The cell surface differentiation markers for M1 macrophages are CD11c, CD44, CD163, CD172, while those for M2 are arginase 1, CD206, and CD301^28^. In the initial studies, the lean mice demonstrated M2 dominant phenotype in ATMs and the obese mice demonstrated M1 dominant phenotype, although mixed M1/M2 phenotypes were reported in later studies^29^. M1 ATMs were localized to crown-like structures surrounding adipocytes and released high levels of proinflammatory cytokines such as IL-6, IL-8, and TNF-α. Th1 cells are induced by IL-12 and IFN-γ, and secrete the proinflammatory cytokines IFN-γ and TNF-α, which promote the differentiation of M1 macrophages^28^. Gal-9 is abundantly expressed and remained in cytoplasm in steady states; however, it is secreted out as DAMPs or PAMPs and induces prominent apoptosis of Th1 and Th17 cells by binding to Tim-3 as a ligand. Thus, we initially hypothesized that the deficiency of Gal-9 in diet-induced obesity (DIO) mice would enhance the inflammation process in visceral adipose tissues associated with deteriorated obesity phenotype and insulin resistance. Unexpectedly, Gal-9^−/−^ DIO mice demonstrate improved obesity and insulin resistance phenotypes without alterations in adipose tissue inflammation and M1/M2 polarization. Similarly, unexpected results were observed in a pristane-induced lupus model in Gal-9^−/−^ BALB/c mice^30^. Since the injection of recombinant Gal-9 induced the apoptosis of T cells and ameliorated systemic lupus erythematosus (SLE) in MRL-*lpr* lupus-prone mice^20^, we thought that Gal-9^−/−^ BALB/c mice would demonstrate the aggravation of the disease. However, pristane treated Gal-9^−/−^ BALB/c mice were protected from nephritis, arthritis and peritoneal lipogranuloma formation^30^. The commercially available ELISA kits for Gal-9 detected the degradation products and measured concentrations were higher than actual concentrations of intact Gal-9. By the development of specific ELISA for whole and intact Gal-9, the concentration in healthy human subjects was 110 pg/mL (67–154 pg/mL)^31^. Although Gal-9 is abundantly and ubiquitously expressed in various organs, the physiological plasma concentration of Gal-9 is very low. The apoptotic potential of Gal-9 was mainly demonstrated by the application of recombinant Gal-9 protein and it required much higher doses at pharmacological range above the physiological concentrations. Furthermore, the cross-link and lattice formation of the cell-surface glycoproteins, and the subsequent intracellular signaling by Gal-9 may be brought by the optimum concentrations of Gal-9, but not by excess amount of Gal-9^32,33^. Thus, the Gal-9 released from adipocytes in obesity may not be enough to demonstrate the potential to induce the apoptosis Th1 cells in adipose tissues.

In adipose tissues in DIO mice, the expression of Gal-1, Gal-9 in subcutaneous adipose tissues (SAT), and Gal-3 in SAT and visceral adipose tissues (VAT) were progressively increased. In contrast, Gal-12 declined overtime in VAT of DIO mice^34^. Although these galectins were expressed in both mature adipocytes and SVF, Gal-1 increased in adipocytes, Gal-3 and Gal-9 increased in SVF, whereas Gal-12 was dominant in adipocytes^34^. Gal-1^34^ ablation resulted in increased adiposity with impaired glucose metabolism and systemic inflammation. Gal-3 deficiency was reported to link to increased adiposity and dysregulated glucose metabolism^35^ in the initial study; however, Gal-3 deletion resulted in the improvement of insulin resistance and hematopoietic-derived Gal-3 was shown to cause cellular and systemic insulin resistance^36^ in the later study. The ablation of Gal-12 known as a negative regulator of lipolysis resulted in increased mitochondrial respiration, reduced adiposity and increased insulin sensitivity^37^, while the ablation of Gal-12 was also characterized by the M2 polarization associated with reduced insulin sensitivity in cultured macophages^38^.

The role of secreted Gal-9 in extracellular milieu has been extensively investigated by focusing on the modulation of immune function, *i.e.* the regulation of apoptosis and immune checkpoint; however, the cytosolic function of Gal-9 remains unexplored especially in the aspect of redox state of the cells^39^. It is well known that galectins rapidly lose sugar-chain binding activities if they are not kept in reducing buffers because of cross-linking and oxidation of cysteine residues or tryptophan residue in the carbohydrate recognition domain. The labile galectins are Gal-1 and Gal-2, while most others, such as Gal-3 and Gal-4, are more stable in the absence of reducing conditions. Since galectins are synthesized in the cytosol, which is highly reducing environment, sugar-chain binding activities are maintain in the cytosol. The oxidation of Gal-1 promoted the formation of the Cys16-Cys88 disulfide bond, and multimers through Cys2 and oxidized Gal-1 did not bind to lactose^40^. Interestingly, oxidized form of Gal-1 without lectin activity promotes the axonal regeneration, not in the reduced form^41^. Similarly, the oxidation of Gal-2 by H_2_O_2_ resulted in the loss of lectin activity, while treatment of Gal-2 with S-nitrosocysteine prevented H_2_O_2_-induced inactivation^42^. In contrast to Gal-1 and Gal-2, Gal-3 is actively involved in the regulation of redox state in the patients with aortic stenosis (AS) and animal model of cardiac damage. Gal-3 was up-regulated in myocardial biopsy from AS patients and negatively correlated with the expression of PRDX4^43^. The inhibition Gal-3 with modified citrus pectin (MCP) exhibited dramatic improvement in cardiac function of the doxorubicin-treated rats by upregulating anti-oxidant PRDX4^44^.

Although the involvement of Gal-9 in redox regulation had been totally unknown, the role Gal-9 in cell surface redox control of T cells was reported^25^. Thioredoxin and protein disulfide isomerase (PDI) are members of PDI family. PDI cleaves disulfide bonds by thiol/disulfide exchange on the plasma membrane, while PDI in the endoplasmic reticulum operates as an oxidase, creating disulfide bonds^25^. Exogenous Gal-9 binds to PDI via *O*-glycan, maintains the retention of PDI on surface of T cells, and increases free thiols in the disulfide/thiol cycles^45,46^. In our experiments, we firstly demonstrated that Gal-9 binds to PRDX2 independent of sugar-binding activity of Gal-9, since the binding was not cancelled by the addition of lactose. Two pairs of cysteine residues in PRDX2 form disulfide-linked homodimers in the presence of H_2_O_2_, and they return to their monomers by thioredoxin and thioredoxin oxidase system^47^. Higher PRDX2 dimer/monomer ratio indicates the oxidative stress and is a sensitive sensor for redox states in the cytosol. In 3T3L1 adipocytes, the knockdown of Gal-9 resulted in lower PRDX2 dimer/monomer ratio in the presence of H_2_O_2_, suggesting the amelioration of oxidative stress in cytosol. Furthermore, it can be speculated that the disruption of the binding between Gal-9 and PRDX2 by knocking down Gal-9 resulted in the facilitation of dimer to monomer conversion by thioredoxin　(Supplementary Fig. 6). The oxidative stress induces the insulin resistance associated with increased levels of inflammatory cytokines, such as leptin, MCP-1, IL-6, and TNF-α, and recued levels of adiponectin^48^. In Gal-9^−/−^ C57BL/6J mice, they were resistant to diet-induced obesity and the absence of Gal-9 ameliorated the oxidative stress by shifting lower dimer/monomer ratio of PRDX2 in 3T3L1 adipocytes.

In conclusion, Gal-9^−/−^C57BL/6J mice fed with HFHS chow were resistant to DIO without the alterations in production of inflammatory cytokines, M1/M2 macrophage polarization, and formation of crown-like structure. Although recombinant Gal-9 is known to induce the apoptosis of Th1 cells and reduction of M1 macrophages, the reduction of body weight was independent of BMCs revealed by BMT experiments. Instead, we found that Gal-9 binds to PRDX2 and demonstrated that Gal-9 knockdown ameliorated the oxidative stress and reduced dimer/monomer ratio of PRDX2 in adipocytes. The inhibition of Gal-9 in adipocytes may be a new therapeutic approach targeting the oxidative stress and subsequent insulin resistance in obesity.

## Methods

### Animals

*Lgals9*^tm1Glp^/*Lgals9*^tm1Glp^ (Gal-9^−/−^) C57BL/6J mice were kindly provided by GalPharma Co. Ltd. Gal-9^−/−^ BALB/c mice were crossed to C57BL/6JJcl mice (CLEA Japan, Tokyo, Japan) for 10 generations. Gal-9^-^ was detected by PCR primers 5′-GCGAGGCCAGAGGCCACTTGTGTAGC-3′ and GTGACAATACTGTTCCTCTGCAGG-3′, while *Lgals9*^wt^ (Gal-9^wt^) by 5′-TGGGGTGTCCTGCAGACAGCACATAA-3′ and 5′-CCAGTGCTACGGCGACATAGCCTC-3′. By crossing Gal-9^wt/-^ C57BL/6JJcl mice, we produced Gal-9^wt/wt^, Gal-9^wt/-^, and Gal-9^−/−^ littermates by standard breeding techniques. Gal-9^wt/wt^ and Gal-9^−/−^ mice were used for the following experiments. The 6-week-old male mice were fed with standard chow diet (STD) (MF, Oriental Yeast, Japan) or high fat-high sucrose diet (HFHS) (D12331, Research Diets, New Brunswick, NJ), and they were euthanized at 28 weeks of age. $\dot{V}$˙O_2_ and RQ were continuously monitored for 24 h by using O_2_/CO_2_ metabolism measuring system (MK-5000, Muromachi, Kyoto, Japan). The locomotor activity was recorded for 24 h by the frequency of interrupting an infrared sensor (ACTIMO-100 N, SHINFACTORY, Fukuoka, Japan). IPGTT was performed at 20–22 weeks of age after 12 h fasting, blood glucose and plasma insulin levels were measured (Skylight Biotech, Tokyo, Japan). At 28 weeks of age, sera and tissue samples were collected and weighed after 12 h fasting. Serum leptin, adiponectin (ELISA), cholesterol, triglyceride, non-esterified free fatty acid (HPLC), liver cholesterol and liver triglyceride (Folch’s method) were measured by Skylight Biotech, Tokyo. All animal experiments were approved by the Animal Care and Use Committee of the Department of Animal Resources, Advanced Science Research Center, Okayama University. All animal experiments were performed in accordance with relevant guideline and regulations.

### Immunofluorescence

Epididymal adipose tissues were first stained with rat anti-mouse F4/80 and rabbit anti-mouse perilipin, and subsequently stained with goat anti-rat IgG (Alexa Fluor 488) and donkey anti-rabbit IgG (Alexa Fluor 555), respectively (Invitrogen). Nuclear stain was performed by DAPI.

### Quantitative real-time PCR

Total RNAs from epididymal fat tissue were extracted from using RNeasy Lipid Tissue Mini Kit (Qiagen), cDNAs were generated using the High Capacity cDNA Reverse Transcription Kit (Applied Biosystems). Quantitative real-time PCR was performed by using Step One Plus Real-Time PCR System (Applied Biosystems), Universal Master Mix II (Life Technologies) and TaqMan Gene Expression Assays with specific primers; *Adgre1* (Mm00802529_m1), *Arg1* (Mm00475988_m1), *Il6* (Mm00446190_m1), *Il10* (Mm001288386_m1), *Il18* (Mm00434225_m1), *Mrc1* (Mm00485148_m1), *Nos2* (Mm00440502_m1), *Tnf* (Mm00443258_m1), and *Ym1* (*Chil3*) (Mm00657889_m1).

### Flow cytometry analysis

Mature adipocytes and stromal vascular fractions (SVF) were isolated as described below. Epididymal fat tissues were minced and incubated in a fresh digesting media of Krebs Ringer HEPES (KRH) buffer for 60 min at 37 °C and were separated into adipocytes and SVF by using mesh with a grid diameter 300 µm. The SVF cells were subjected flow cytometry analysis as previously described^30^. The cells were first incubated at 4 °C for 10 min with Purified Rat Anti-Mouse CD16/CD32 (Mouse BD Fc Block) (BD Pharmingen) to reduce nonspecific binding of antibodies to FcR receptors. The cells (1 × 10^6^) derived from epididymal fat tissues were incubated at 4 °C for 30 min in Stain Buffer (BD Pharmingen) with the relevant optimized amount of fluorochrome conjugated antibodies or the appropriate isotype controls: PerCP-Cy 5.5 Rat Anti-Mouse CD11b, BV421 Hamster Anti-Mouse CD11c, PE-Cy 7 Mouse Anti-Mouse CD45.2 (BD Pharmingen), Rat Anti-Mouse CD206 Alexa Fluor 647 (AbD Serotec), and Anti-Mouse F4/80 Antigen FITC (eBioscience). Dead cells were excluded from analysis using 7-aminoactinomycin D staining (BD pharmigen). All data were acquired with FACSAria I flow cytometer (BD Biosciences) and analysed using FlowJo software (TreeStar, Ashland, OR).

### Bone marrow transplantation

Mice received bone marrow transplantation by the standard protocols as previously described^49^. In brief, the donor bone marrow cells were obtained from the long bones of 10–12 weeks old Gal-9^wt/wt^ (n = 3) and Gal-9^−/−^ (n = 2) C57BL/6JJcl mice. T-cell depletion was performed with anti-CD90.2 Microbeads and an AutoMACS system (Miltenyi Biotec, Bergisch Gladbach, Germanry) according to the manufacturer’s instructions. The recipient 4–6 weeks old Gal-9^wt/wt^ (n = 6) and Gal-9^−/−^ (n = 6) C57BL/6JJcl mice were given 5.5 Gy whole body irradiation twice. Then, the recipient mice were injected with 5 × 10^6^ T cell-depleted bone marrow cells from donors. The mice were fed with HFHS for additional 18 weeks.

### Immunoprecipitation and nanoLC-MS/MS analysis

Epididymal fat tissues (200 mg each) derived from Gal-9^wt/wt^ C57BL/6JJcl mice were minced, lysed in 500 μl of RIPA buffer (Thermo Scientific) containing 1 mM DTT under reducing condition, and immunoprecipitated with Protein G Immunoprecipitation kit (Sigma) by using anti-galectin-9 (Biolegend, Cat#137,902) or Purified rat IgG2a, κ isotype control (Biolegend, Cat#400,502) antibodies. The immunoprecipitated protein complexes were electrophoresed in SuperSep Ace 15–20% Tricine Gel (GE Healthcare), and gel-digested with trypsin. The samples were lyophilized, suspended in 20 μl of 0.1% formic acid, and subjected to nanoLC-MS/MS using UltiMate 3000 HPLC, Q-Exactive Plus, and Xcalibur (Thermo Scientific). The proteins were searched and identified by Mascot (Matrix Science, Boston, MA). The scores by Mowse scoring algorithm, hit peptide fragment numbers, the exponentially modified protein abundance index (emPAI) were analysed. The gene annotation was performed by PANTHER 15.0 (http://www.pantherdb.org/).

The lysates from epididymal fat tissues of Gal-9^wt/wt^ and Gal-9^−/−^ C57BL/6JJcl mice were also immunoprecipitated with anti-peroxiredoxin 2 (PRDX2) antibody (abcam, Cat#ab109367), and subjected with Western blot analysis with anti-galectin-9 antibody (Biolegend, Cat#137,902).

### Pull-down assay

Full-length cDNA of peroxiredoxin 2 was amplified by primers; 5′-GGGGGGCTCGAGATGGCCTCCGGCAACGCGCA-3′ and 5′-GGGGGGGGATCCTCAGTTGTGTTTGGAGAAGT-3′. The PCR products were digested with restriction enzymes, *Xho*I and BamHI, and ligated to FLAG-HA-pcDNA3.1 (Addgene, Plasmid #52,535). The plasmid (PRDX2-FLAG-HA-pcDNA3.1) was transfected 3T3L1 cells and lysed in RIPA buffer (Thermo Scientific) containing 1 mM DTT in the presence and absence of 0.2 M lactose. HA-tagged protein purified by HA-tagged Protein Purification Kit (MBL, Tokyo, Japan). They were subjected to SDS-PAGE with 1 mM DTT under reducing condition, and Western blot analysis using anti-galectin-9 antibody (Biolegend, Cat#137,902), anti-peroxiredoxin 2 (PRDX2) antibody (abcam, Cat#ab109367), anti-thioredoxin (proteintech, Cat#14,999–1-AP), and anti-HA antibody (MBL, Cat#561).

### 3T3L1 culture and siRNA experiments

3T3L1 cells (ATCC) were cultured in Dulbecco's Modified Eagle's Medium (Thermo Fisher Scientific) containing 10% fetal bovine serum and they were transfected with Silencer select Pre-designed siRNA Lgals9 (Gal-9 siRNA) and silencer select negative control siRNA (NC siRNA) (Thermo Fisher Scientific, Cat#s69189) by Lipofectamine RNAi MAX (Cat#13,778,030). After 40 h, the 3T3L1 cells were further cultured for 20 minutes in the absence and presence of 10 μM H_2_O_2_.

The cells were lysed in 500 μl of RIPA buffer and subject to SDS-PAGE under non-reducing conditions and Western blot analysis using anti-galectin-9 antibody (Biolegend, Cat#137,902), anti-peroxiredoxin 2 (PRDX2) antibody (abcam, Cat#ab109367), anti-GAPDH antibody (Cell Signaling Technology, Cat#8884), anti-phospho-p38 MAPK (Thr180/Try182) antibody (Cat#9211), anti-p38 MAPK antibody (Cat#9212), anti-phospho-SAPK/JNK (Thr183/Try185) antibody (Cat#9251), anti-SAPK/JNK antibody (Cat#9252), anti-phospho-p44/42 MAPK (Erk1/2)(Thr202/Try204) antibody (Cat#9101), and anti-p44/42 MAPK (Erk1/2) antibody (Cat#9101).

### Statistical analysis

All results are expressed as means ± standard deviation (SD). Normal distribution was confirmed by Shapiro–Wilk test. Only plasma insulin concentration and percentage of M1 and M2 macrophage did not follow the normal distribution. For parametric analyses, the multiple comparisons were performed by one-way ANOVA with Tukey–Kramer method and two-pair comparisons by Student’s *t* test using SPSS software (IBM, Chicago, IL). For non-parametric analyses, the multiple comparisons were performed by Kruskal–Wallis test with Bonferroni correction. A value of *p* < 0.05 was regarded as statistically significant.

## Supplementary Information

Supplementary Information 1.

Supplementary Information 2.

Supplementary Information 3.
