# Author Correction: *Lgals*9 deficiency ameliorates obesity by modulating redox state of PRDX2

**DOI:** 10.1038/s41598-021-98293-1

**Published:** 2021-09-14

**Authors:** Tomokazu Nunoue, Satoshi Yamaguchi, Sanae Teshigawara, Akihiro Katayama, Atsuko Nakatsuka, Jun Eguchi, Toshiro Niki, Jun Wada

**Affiliations:** 1 Department of Nephrology, Rheumatology, Endocrinology and Metabolism, Okayama University Graduate School of Medicine, Dentistry and Pharmaceutical Sciences, 2‑5‑1 Shikata‑cho, Kita‑ku, Okayama, 700‑8558 Japan; 2 Department of Immunology, Kagawa University, Takamatsu, Kagawa Japan

Correction to: *Scientific Reports* 10.1038/s41598-021-85080-1, published online 16 March 2021

The original version of this Article contained errors.

In Figure 5, the size marker gel image was mistakenly placed in Panel (e). The original Figure 5 and accompanying legend appear below.

**Figure 5:**
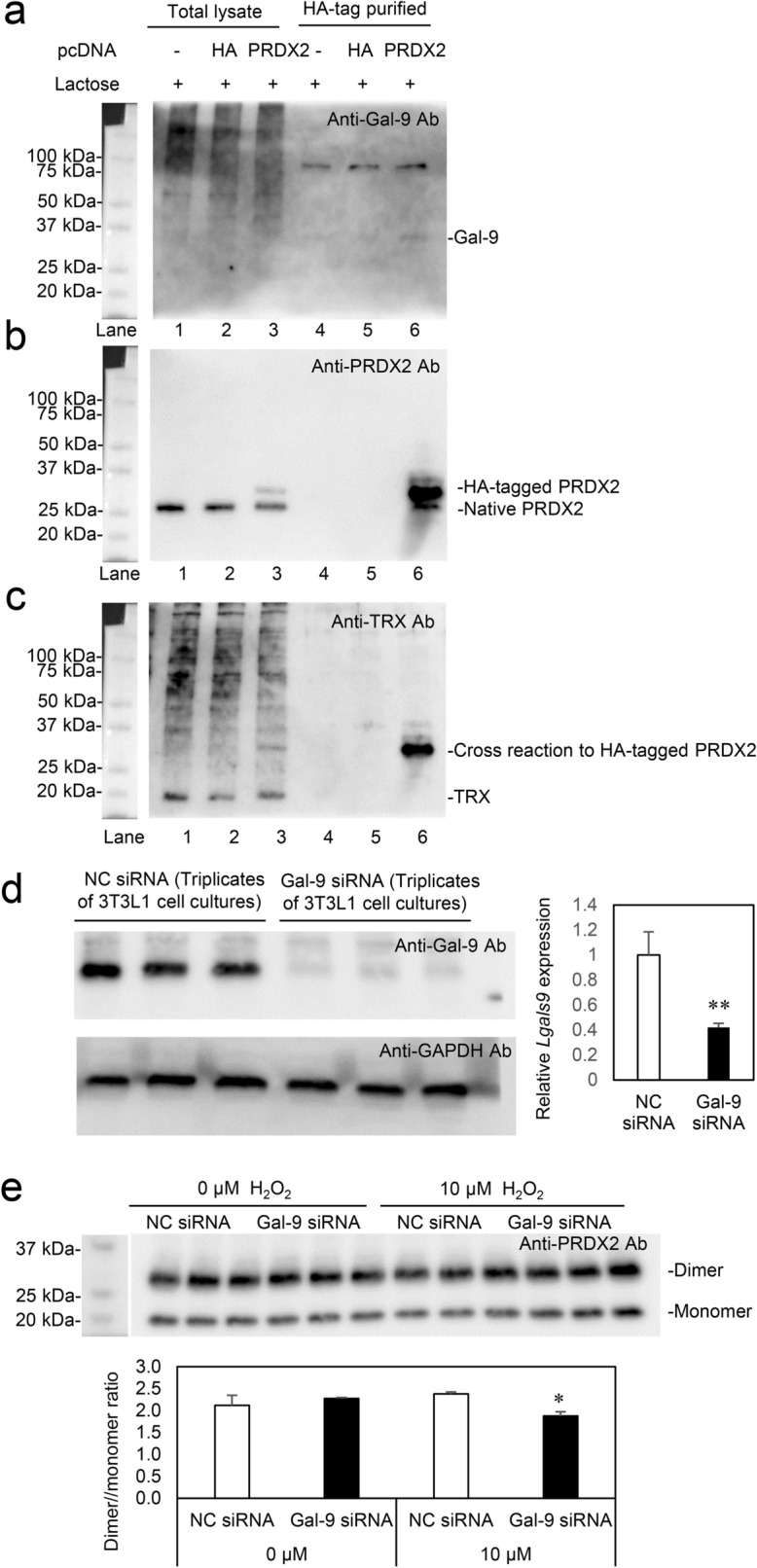
Pull-down assay and Gal-9 siRNA experiments in 3T3L1 cells. (**a**) PRDX2-FLAG-HA-pcDNA3.1 (PRDX2) and FLAG-HA-pcDNA3.1 (HA) were transfected into 3T3L1 cells. In the presence of 0.2 M lactose, the protein complexes were HA-tag purified with Anti-HA tag Beads, and subjected to SDS-PAGE under reducing conditions and Western blot analysis. The membrane was incubated with anti-Gal-9 antibody. (**b**) The membrane was stripped off and incubated with anti-peroxiredoxin 2 (PRDX2) antibody. (**c**) The membranes was again stripped off and incubated with anti-thioredoxin (TRX) antibody. (**d**) 3T3L1 cells were treated with Silencer select Pre-designed siRNA Lgals9 (Gal-9 siRNA) and Silencer select negative control siRNA (NC siRNA) for 40 h. (**e**) After the treatment of 3T3L1 cells with siRNAs, the cells were further cultured in the absence and presence of 10 μM H_2_O_2_ for 20 min. *, *p* < 0.05; **, *p* < 0.01. Two-pair comparisons by Student’s *t* test.

Additionally in Supplementary Figure 4 and 5, the panel labels and corresponding red boxes for uncropped images were omitted. The original Supplementary Information 1 file is provided below.

The original Article and accompanying Supplementary Information 1 file have been corrected.

## Supplementary Information

Supplementary Information 1.

