# Supplementary material for: Author Correction: Lgals9 deficiency ameliorates obesity by modulating redox state of PRDX2
Source: Sci Rep. 2021 Sep 14;11:18611. doi: 10.1038/s41598-021-98293-1 (PMC8440629; doi:10.1038/s41598-021-98293-1)
Supplement: Supplementary file 1 — Supplementary Information 1. [file 41598_2021_98293_MOESM1_ESM.pdf]

Supplementary Figure 1

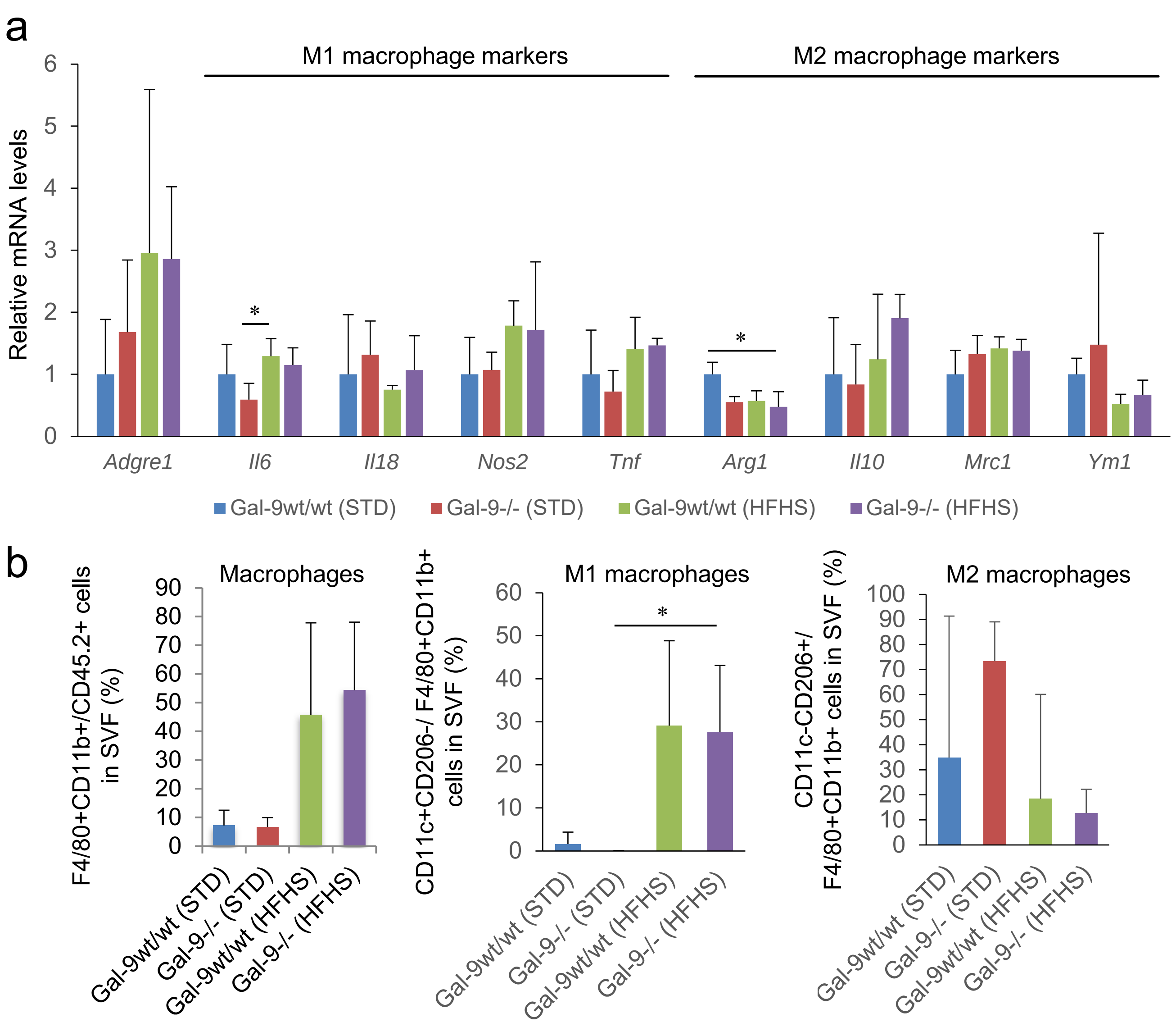

Supplementary Figure 1. Quantitative RT-PCR using total RNA isolated from epididymal adipose tissues and FACS analyses for M1 and M2 macrophages.

a. Quantitative RT-PCR for M1 and M2 macrophage markers using total RNA isolated from epididymal adipose tissues. Gal-9$^{wt/wt}$ (STD), n=2; Gal-9$^{-/-}$ (STD), n=3, Gal-9$^{wt/wt}$ (HFHS), n=4; Gal-9$^{-/-}$ (HFHS), n=4 in *Adgre1*. Gal-9$^{wt/wt}$ (STD), n=2; Gal-9$^{-/-}$ (STD), n=4, Gal-9$^{wt/wt}$ (HFHS), n=4; Gal-9$^{-/-}$ (HFHS), n=4 in *Il6*. Gal-9$^{wt/wt}$ (STD), n=2; Gal-9$^{-/-}$ (STD), n=3, Gal-9$^{wt/wt}$ (HFHS), n=3; Gal-9$^{-/-}$ (HFHS), n=4 in *Il18*, *Nos2, Arg1*, *Mrc1*, and *Ym1*. Gal-9$^{wt/wt}$ (STD), n=4; Gal-9$^{-/-}$ (STD), n=4, Gal-9$^{wt/wt}$ (HFHS), n=3; Gal-9$^{-/-}$ (HFHS), n=4 in *Tnf*. Gal-9$^{wt/wt}$ (STD), n=4; Gal-9 -/- (STD), n=4, Gal-9$^{wt/wt}$ (HFHS), n=3; Gal-9 -/- (HFHS), n=3 in *Il10*. *, p<0.05. **b**. Percentage of F4/80+CD11b+/CD45.2+ (macrophages), CD11c+CD206-/F4/80+CD11b+ (M1 macrophages), and CD11c-CD206+/F4/80+CD11b+ (M2 macrophages). Gal-9$^{wt/wt}$ (STD), n=3 Gal-9$^{-/-}$ STD), n=3, Gal-9$^{wt/wt}$ (HFHS), n=2; Gal-9$^{-/-}$ (HFHS), n=3. *, p<0.05. One-way ANOVA with Tukey–Kramer.

Supplementary Figure 2

IP: Peroxiredoxin 2 (PRDX2)
Blot: anti-galectin-9 (Gal-9)

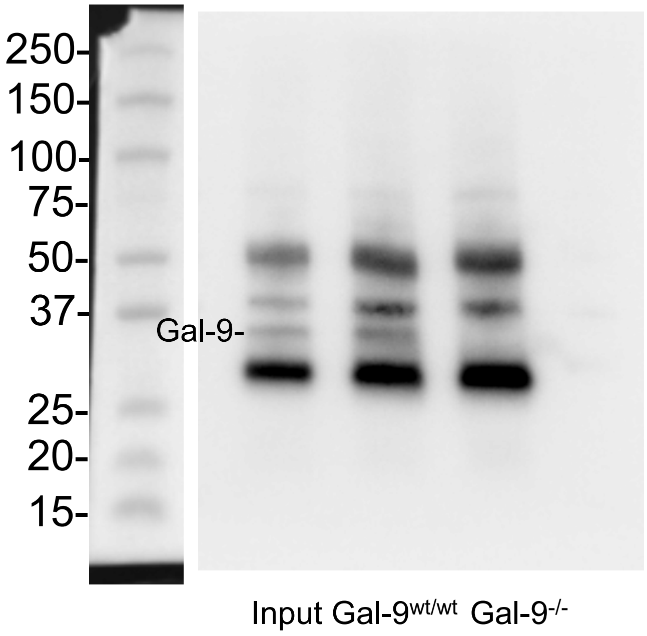

Epididymal fat

Supplementary Figure 1. Immunoprecipitation with anti-peroxiredoxin (PRDX2) antibody and blotted with anti-galectin-9 (Gal-9) antibody.

Supplementary Figure 3

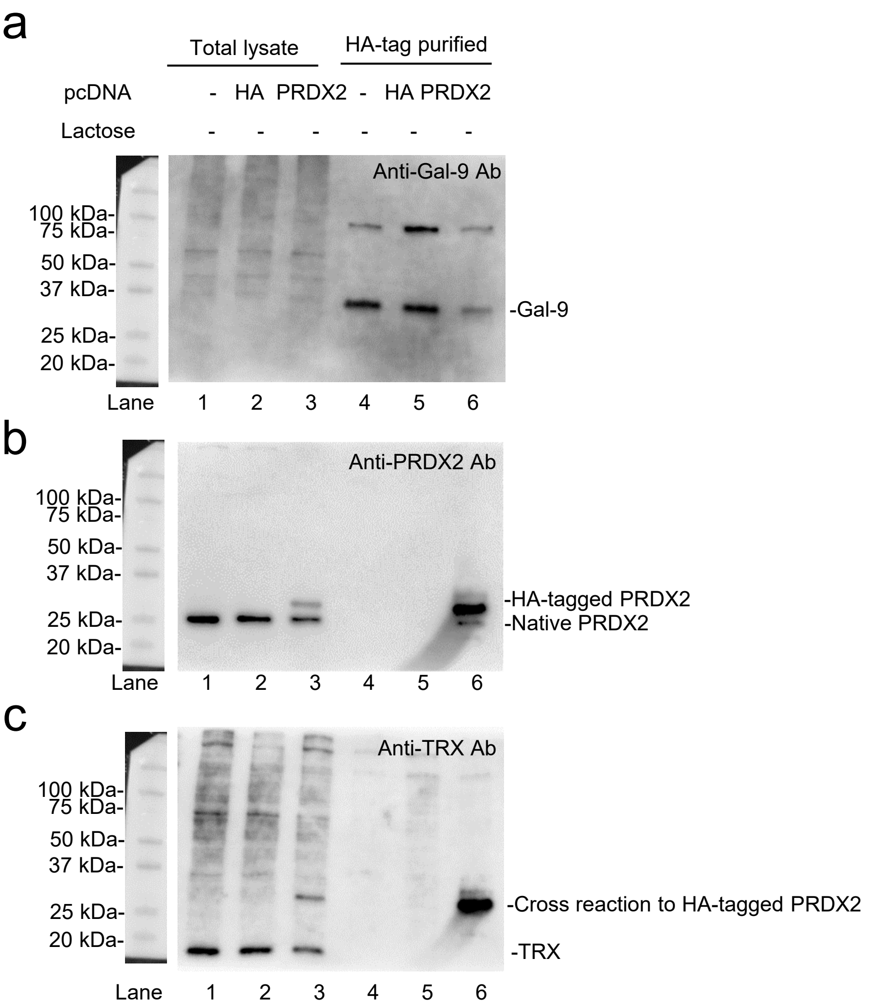

Supplementary Figure 3. Pull-down assay and Gal-9 siRNA experiments in 3T3L1 cells.

a. PRDX2-FLAG-HA-pcDNA3.1 (PRDX2) and FLAG-HA-pcDNA3.1 (HA) were transfected into 3T3L1 cells. In the absence of 0.2 M lactose, the protein complexes were HA-tag purified with Anti-HA tag Beads, and subjected to SDS-PAGE under reducing conditions and Western blot analysis. The membrane was incubated with anti-Gal-9 antibody. **b**. The membrane was stripped off and incubated with anti-peroxiredoxin 2 (PRDX2) antibody. **c**. The membranes was again stripped off and incubated with anti-thioredoxin (TRX) antibody.

Supplementary Figure 4

a

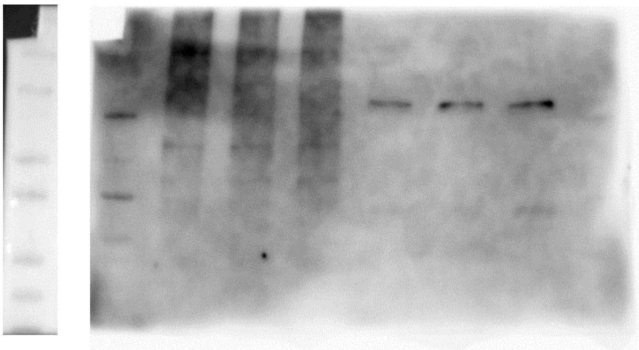

b

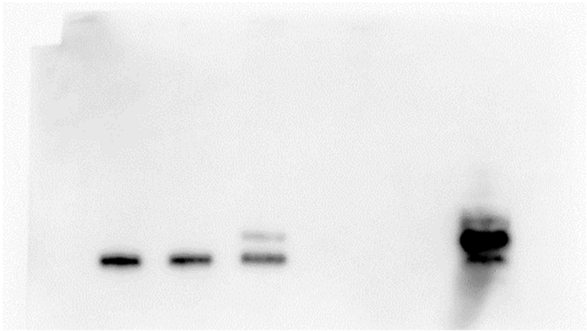

c

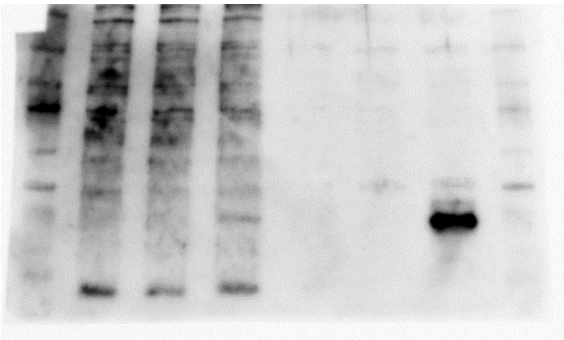

d

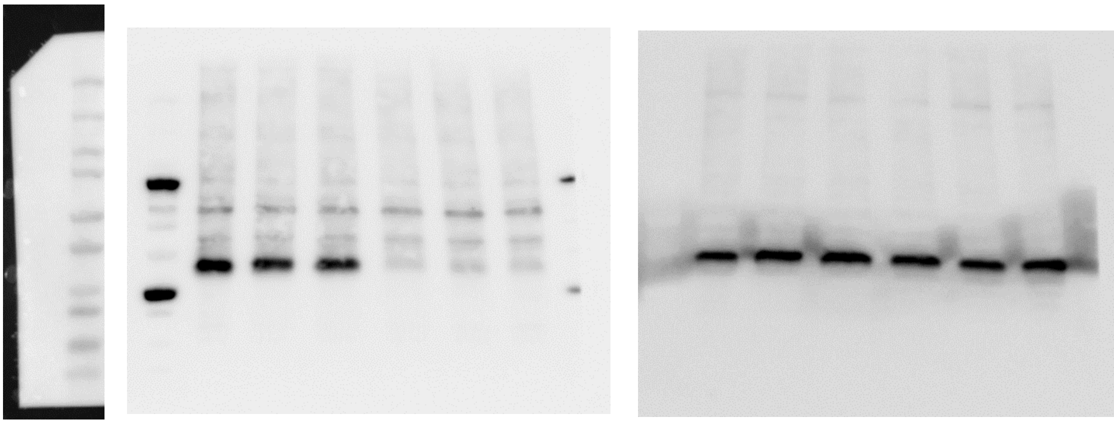

e

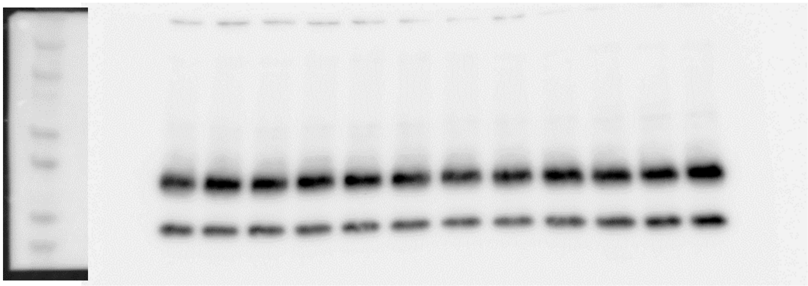

Supplementary Figure 4. Uncropped gel images

Supplementary Figure 5

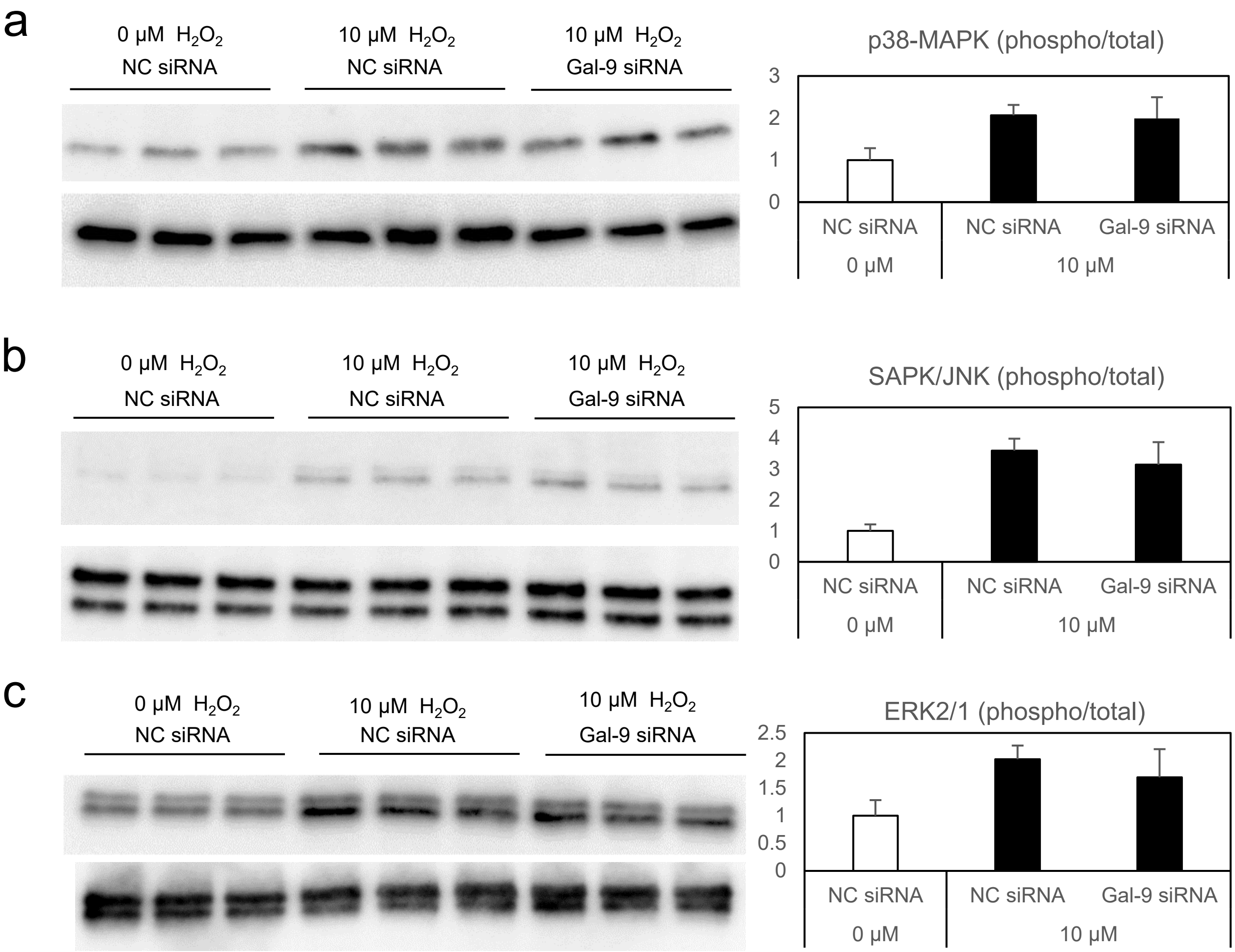

Supplementary Figure 5. **Gal-9 siRNA experiments in 3T3L1 cells and Western blot analyses for oxidative stress signaling pathways.**

3T3L1 cells were treated with Silencer select Pre-designed siRNA Lgals9 (Gal-9 siRNA) and Silencer select negative control siRNA (NC siRNA) for 40 hours. After the treatment of 3T3L1 cells with siRNAs, the cells were further cultured in the absence and presence of 10 μM H_2O_2 for 20 minutes. Western blot analyses for the oxidative stress signaling molecules such as p38 mitogen-activated protein kinase (p38-MAPK) (**a, d**), stress-activated protein kinase (SAPK)/Jun amino terminal kinase (JNK) (**b, e**), and p42/p44 mitogen-activated protein kinases [MAPK; extracellular signal-regulated kinase 2/1 (ERK2/1)] (**f**, **g**) were performed. *, p<0.05; **, p<0.01. Two-pair comparisons by Student's *t* test.

Supplementary Figure 5

d

Uncropped images
phospho p38-MAPK

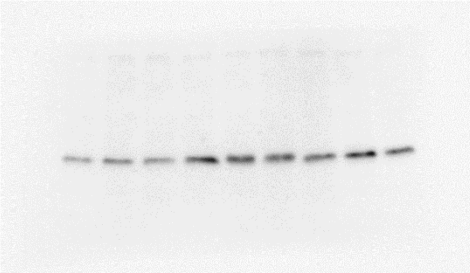

Uncropped images
p38-MAPK

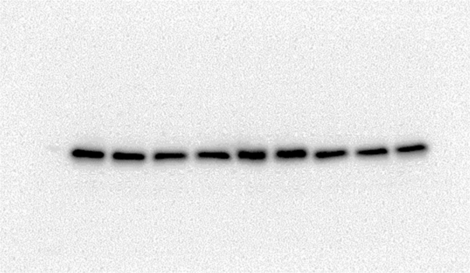

e

Uncropped images
phospho SAPK/JNK

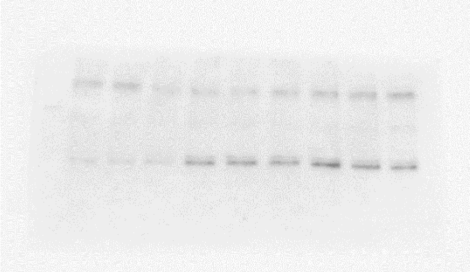

Uncropped images
SAPK/JNK

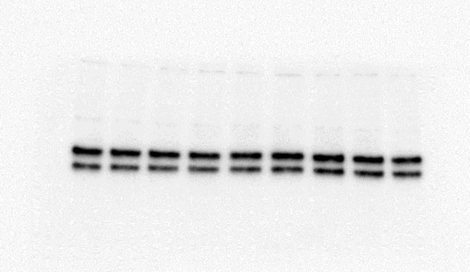

f

Uncropped images
phospho ERK2/1

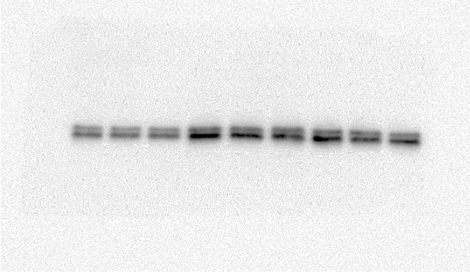

Uncropped images
ERK2/1

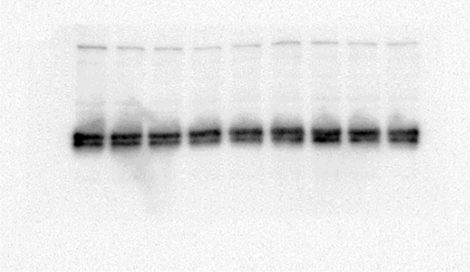

Supplementary Figure 6

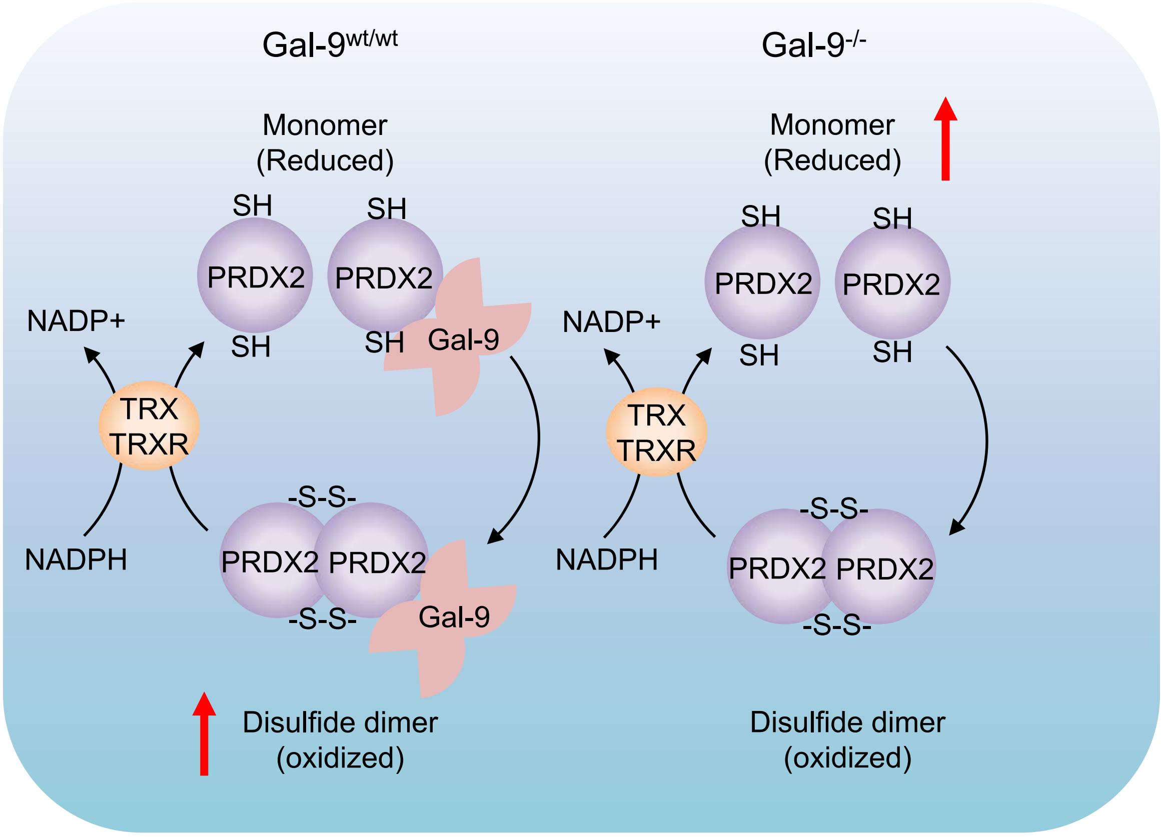

Supplementary Figure 6. Interaction of peroxiredoxin (PRDX2) and galectin-9 (Gal-9) in Gal-$9^{wt/wt}$ and Gal-$9^{-/-}$ mice. TRX, thioredoxin; TRXR, thioredoxin reductase.
